# Is *Toxoplasma gondii* infection correlated with nonalcoholic fatty liver disease?- a population-based study

**DOI:** 10.1186/s12879-018-3547-1

**Published:** 2018-12-06

**Authors:** Jiaofeng Huang, Haoyang Zhang, Shiying Liu, Mingfang Wang, Bo Wan, Bharat Velani, Yueyong Zhu, Su Lin

**Affiliations:** 1 Department of Liver Research Center, the First Affiliated Hospital of Fujian Medical University, Fuzhou, No. 20, Chazhong Road, Taijiang District, Fuzhou, 350001 Fujian China; 2 School of Public Health, Sun Yat-sen University, Guangzhou, 350002 Guangdong China; 3 Faculty of Life Sciences and Medicine, King’s College London, London, SE1 1UL UK; 4 Basildon and Thurrock University Hospitals NHS Foundation Trust, Nethermayne, Basildon, Essex SS16 5NL UK

**Keywords:** Non-alcoholic fatty liver, *Toxoplasma gondii*, NHANES

## Abstract

**Background:**

Previous studies have suggested that *Toxoplasma gondii* (*T. gondii*) infection might be associated with fatty liver disease. However, the relationship between non-alcoholic fatty liver disease (NAFLD) and *T. gondii* infection has not been investigated in a large population. We aimed to study the relationship between those two diseases using a population-based dataset from the United States.

**Methods:**

The data were collected from the third National Health and Nutrition Examination Survey (NHANES III) between 1988 and 1994. Statistical analysis was applied to compare the prevalence of NAFLD in anti-*T. gondii* antibody-positive participants with antibody-negative ones.

**Results:**

A total of 9465 persons with a mean age of 44.33 ± 16.21 years, 46.9% of which were males, were included in the final analysis. Their mean BMI was 27.60 ± 5.96 kg/m^2^. A total of 2520 participants (26.62%) were positive for the *T. gondii* antibody. There was an increasing trend of seroprevalence of *T. gondii* with age (P for trend < 0.001). The incidence of NAFLD in the seropositive group was higher than that in the seronegative group (27.10% vs 23.40%, *p* < 0.001). In addition to this, metabolic biomarkers, including serum lipid, fasting blood-glucose, and uric acid were also significantly higher in the seropositive group. However, multivariate analysis revealed that *T. gondii* infection was not an independent risk factor for NAFLD. Age was independently correlated with both the prevalence of *T. gondii* and NAFLD.

**Conclusions:**

Patients with *T. gondii* infection may have a higher prevalence of NAFLD. Age may have an effect on the increase of NAFLD in the *T. gondii* seropositive population.

## Background

*Toxoplasma gondii (T. gondii)* infection is a major global public health problem. Approximately 30% of the world’s population show serological evidence of infection [1]. Although most infections are subclinical and benign, some may cause severe consequences, including lymphadenopathy, hepatitis, ophthalmitis, schizophrenia and other important organ dysfunction [2, 3]. When the liver is involved, *T. gondii* infection can present with hepatomegaly, liver inflammation, liver granuloma formation and cirrhosis [4, 5]. Several studies demonstrated a higher seroprevalence of *T. gondii* antibody in patients with liver cancer, cirrhosis, acute and chronic hepatitis [4–8].

Non-alcoholic fatty liver disease (NAFLD) is a disease with an excessive accumulation of fat in the liver, with different complications including inflammation, fibrosis, cirrhosis and hepatocellular carcinoma. It is the most common chronic liver disease with a global prevalence of 30% [9]. A recent study demonstrated that mice infected with *T. gondii* had significant inflammation and steatosis in the liver [10]. Epidemiological data from eastern China showed that the prevalence rate of *T. gondii* was higher in people with liver steatosis (22.75%) compared to controls (13.86%) [7]. Up to now, this has been the only clinical research looking into the relationship between *T. gondii* infection and NAFLD. However, the data was from eastern China and the sample size was relatively small and poorly representative. It is important to explore this relationship further in a different population with a larger sample size. Determining this association between *T. gondii* and NAFLD may contribute towards the further understanding and control of both diseases. This study analyzes the relationship between *T. gondii* infection and incidence of NAFLD on a population-based dataset from the United States.

## Methods

### Study population

This cross-sectional study was based on the data from The Third National Health and Nutrition Examination Surveys (NHANES III) [11]. The NHANES III is a periodic survey conducted by the National Center between 1988 and 1994, and is the only survey that contains data with both liver ultrasonography examination and anti-*T. gondii* antibodies tests.

Participants who had undergone both ultrasonography examination and serum anti-*T. gondii* antibody tests were selected as cases. Those patients with a history of alcohol consumption and patients with chronic viral hepatitis B or C were excluded.

This study was approved by the Research Ethics Review Board. The informed consents were obtained from all subjects as described in the original research design [12]. All data and further information on NHANES are available on the website [11].

### Demographic variables

Age, sex, and body mass index (BMI) were collected. BMI was calculated as weight (in kilograms) divided by the square of the height (in meters) and classified as underweight (< 18.5 kg/m^2^), normal weight (18.5–24.9 kg/m^2^), overweight (25.0–29.9 kg/m^2^), obese class I (30.0–34.9 kg/m^2^), and obese class II (≥35.0 kg/m^2^) [13].

### Laboratory measurements

Serum cholesterol, triglyceride, urea nitrogen, serum creatinine, fasting blood-glucose (FBG) and uric acid were obtained from original datasets. To be more specific, the blood from each participant was processed, stored and shipped to the Centers for Disease Control and Prevention for further biochemical analysis. All the sera were tested by the same method. The anti-*T. gondii* antibodies were tests using indirect enzyme immunoassay (EIA). The diagnostic threshold was 7 IU/mL as described in the original document. The detailed methods for the testing of anti-*T. gondii* antibodies can be retrieved from the website [14].

### Identification of NAFLD

The level of hepatic steatosis was assessed by reviewing ultrasound video images of hepatic/gallbladder based on the following criteria: 1) the brightness levels of the liver parenchyma, 2) the existence of liver-to-kidney contrast, 3) the existence of deep beam attenuation, 4) the definition of the gallbladder walls, and 5) the existence of echogenic walls in the small intrahepatic vessels [15]. Participants were divided into normal to mild NAFLD, as well as moderate or severe NAFLD according to the results of liver ultrasonography [16].

### Statistical analysis

The categorical variables were expressed as a percentage while continuous data were expressed as mean ± standard deviation. The Chi-square test was used to compare the categorical variable whilst the t-test was used to compare the continuous variables. The potential risk factors of NAFLD were explored using Logistic regression with the enter method. A *P* value < 0.05 in univariate analysis were selected for multivariate analysis. All tests were two-tails and results with a *P* value < 0.05 was considered statistically significant. All analysis was conducted by R 3.4.4 [17].

## Results

### Characteristics of participants

A total of 12,378 participants who received both ultrasound examination and serum tests were eligible. After excluding 2613 individuals with a history of alcohol consumption and 300 cases of viral hepatitis, a total of 9465 persons were included in the final analysis. The mean age of the patients was 44.33 ± 16.21(20–74) years, with 4441 (46.9%) males and 5024 (53.1%) females. The mean BMI of the patients enrolled was 27.60 ± 5.96 kg/m^2^.

### Comparison of *T. gondii* seropositive group and seronegative group

**Fig. 1:**
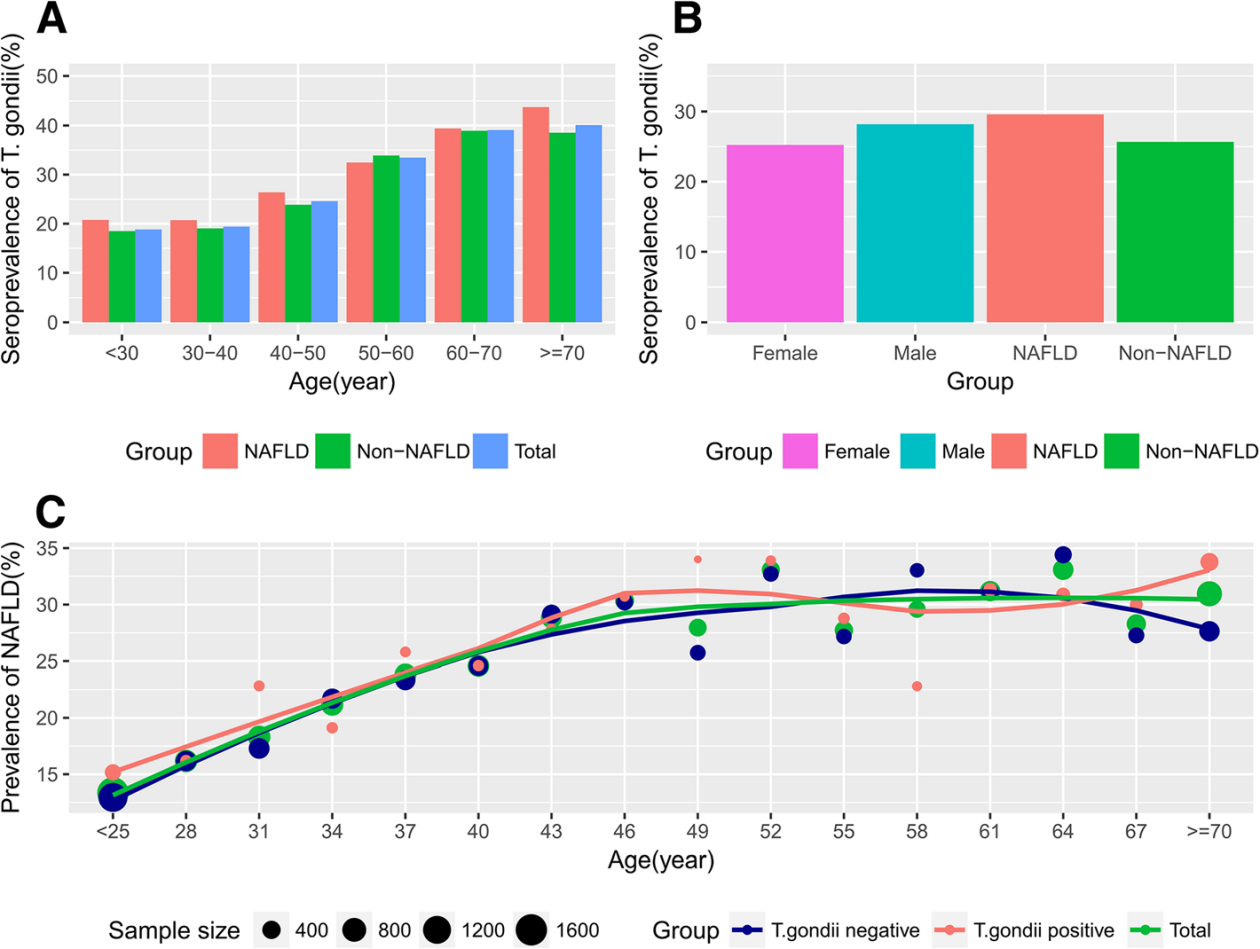
Seroprevalence of *T. gondii* and prevalence of NAFLD. **a** Seroprevalence of *T. gondii* increases with age. In general, the NAFLD group had a slightly higher positive rate of *T. gondii* antibodies than the non-NAFLD group. **b** Seroprevalence of *T. gondii* was higher in the male and NAFLD groups. **c** Prevalence of NAFLD was measured according to age. The scatter plot and its loess fitted line showed a positive association between the prevalence of NAFLD and age. However, the lines representing the *T. gondii* positive group and the *T. gondii* negative group entangle, indicating that there is no significant difference in the prevalence of NAFLD between the two groups after adjusting for age

There were 2520 (26.62%) cases who tested positive for *T. gondii*. The participants were divided into a seronegative group and a seropositive group according to the serological test for anti-*T. gondii* antibody IgG. Males had a higher infection rate than females (28.19% vs. 25.34%, *P* < 0.001) (Fig. 1b). Seropositive participants were older in age than seronegative ones (49.41 years vs. 42.48 years, *P* < 0.001). There was an increasing trend of seroprevalence with age (P for trend < 0.001). The metabolic biomarkers, including cholesterol, triglyceride, FBG, uric acid and glycated hemoglobin (HBA1c) were significantly higher in the seropositive group than the seronegative group (*P* < 0.05). However, most biomarkers for liver cell injury, for example, ALT, AST, and TBIL were comparable between the two groups. Fig. 1a and c show that prevalence of seropositivity for *T. gondii* increased with age. The data showed a higher incidence of NAFLD in the seropositive group than in the seronegative group (27.10% vs 23.40%, *p* < 0.001). Details are shown in Table 1.

**Table 1:** Baseline demographics of the study population

Variables	Anti- *T. gondii* antibody IgG		*t/χ2*	*p* value
	Negative	Positive		
	(*n* = 6945)	(*n* = 2520)		
Age(years)^a^	42.48 ± 15.76	49.41 ± 16.37	−18.71	< 0.001
Age groups (%)				
< 30 years	1982 (28.54%)	460(18.25%)	345.87	< 0.001
30–40 years	1623(23.37%)	391(15.52%)		
40–50 years	1165(16.77%)	380(15.08%)		
50–60 years	839(12.08%)	422(16.75%)		
60–70 years	985(14.18%)	632(25.08%)		
≥70 years	351(5.05%)	235(9.33%)		
Gender (%)				
Male	3189(45.92%)	1252(49.68%)	10.52	0.001
Female	3756(54.08%)	1268(50.32%)		
Ethnicity (%)			43.56	< 0.001
Non-Hispanic white	2375(34.20%)	907(35.99%)		
Non-Hispanic black	2042(29.40%)	696(27.62%)		
Mexican American	2293(33.02%)	761(30.20%)		
Others	235(3.38%)	156(6.19%)		
BMI(kg/m^2^)	27.47 ± 6.00	27.96 ± 5.85	−3.48	< 0.001
NAFLD (%)				
Yes	1625(23.40%)	683(72.90%)	13.77	< 0.001
No	5320(76.60%)	1837(27.10%)		
Cholesterol (mmol/L)^a^	5.38 ± 1.17	5.62 ± 1.20	− 8.53	< 0.001
Triglyceride (mmol/L)^a^	1.64 ± 1.33	1.81 ± 1.42	− 5.27	< 0.001
FBG (mmol/L)^a^	5.47 ± 2.09	5.80 ± 2.52	−6.54	< 0.001
Uric acid (mmol/L)^a^	311.40 ± 92.37	322.18 ± 95.15	− 4.98	< 0.001
HbA1c (%)^a^	5.54 ± 1.15	5.73 ± 1.27	− 6.95	< 0.001
TBIL (mmol/L)^a^	9.92 ± 5.55	9.67 ± 5.00	2.01	0.044
ALT (U/L)^a^	18.32 ± 15.36	17.96 ± 17.44	0.96	0.34
AST (U/L)^a^	21.73 ± 14.09	21.97 ± 14.18	−0.72	0.47
GGT (U/L)^a^	25.72 ± 39.28	28.45 ± 53.48	− 2.69	0.007
ALP (U/L)^a^	86.24 ± 27.85	91.41 ± 38.98	− 7.08	< 0.001
Albumin (g/L)^a^	41.52 ± 3.87	41.16 ± 3.84	4.03	< 0.001
Globulin (g/L)^a^	33.14 ± 4.62	33.72 ± 4.82	− 4.69	< 0.001
Creatinine (umol/L)^a^	93.27 ± 26.13	96.35 ± 32.40	− 4.71	< 0.001

*Abbreviations*: *FBG* fasting blood-glucose, *HBA1c* Glycated hemoglobin, *TBIL* total bilirubin, *ALT* alanine transaminase, *AST* aspartate aminotransferase, *GGT* gamma-glutamyl transpeptidase, *ALP* alkaline phosphatase

^a^Express as mean ± sd or number(%)

### The relationship between NAFLD and *T. gondii* antibody

As shown in Table 1, the prevalence of NAFLD and older age are higher in the seropositive group. However, there was not a statistically significant difference in *T. gondii* antibody seropositivity between NAFLD and non-NAFLD groups in different age groups (*P* > 0.05) (Table 2).

**Table 2:** Prevalence of NAFLD in the different *T. gondii* groups after age stratification

	Non-NAFLD		NAFLD		
	No. *T. gondii* positive	*T. gondii* prevalence	No. *T. gondii* positive	*T. gondii* prevalence	*p value*
Age(years)					
< 30	385	18.50%	75	20.78%	0.31
30–40	300	19.05%	91	20.73%	0.43
40–50	262	23.86%	118	26.40%	0.29
50–60	297	33.90%	125	32.47%	0.62
60–70	435	38.94%	197	39.40%	0.86
≥70	158	38.54%	77	43.75%	0.24

### Multivariate analysis for NAFLD

In order to measure the relationship between *T. gondii* infection and NAFLD, multivariate logistics regression was conducted. Gender, age, BMI, *T. gondii* infection, serum lipid, and FBG were included in the model as independent variables. The model revealed that *T. gondii* infection was not an independent risk factor for NAFLD (OR = 1.01, the *P* value was 0.85). Higher age, FBG, and uric acid were positively associated with the probability of having NAFLD, while the effect of BMI and triglyceride experienced a first fall and then a rise in the positive association. Female (OR = 0.82, *P* value < 0.001) turned to be a protective factor for the onset of NAFLD. See details in Table 3.

**Table 3:** Multivariate logistics analysis for associations between risk factors and NAFLD

Variables	Adjusted OR	*95%CI*	*p* value
Gender			
Male	1	–	–
Female	0.82	0.74–0.91	< 0.001
Age(year)			
< 30	1	–	–
30–50	1.46	1.26–1.68	< 0.001
≥50	1.61	1.39–1.88	< 0.001
BMI(kg/m^2^)			
< 18.5	1	–	–
18.5–30	0.89	0.58–1.37	0.6
≥30	2.4	1.55–3.72	< 0.001
Anti- *T. gondii* antibody IgG			
Negative	1	–	–
Positive	1.01	0.90–1.13	0.85
Cholesterol(mmol/L)			
< 2.9	1	–	–
2.9–6	0.63	0.30–1.33	0.23
≥6	0.66	0.31–1.41	0.29
Triglyceride(mmol/L)			
< 0.45	1	–	–
0.45–1.69	0.75	0.66–0.86	< 0.001
≥1.69	1.68	1.46–1.92	< 0.001
FBG(mmol/L)			
< 3.9	1	–	–
3.9–5.6	1.31	0.78–2.20	0.31
≥5.6	2.41	1.42–4.07	< 0.001
Uric acid(umol/L)			
< 149	1	–	–
149–416	1.66	0.24–0.88	0.12
≥416	2.19	0.66–0.87	0.02

## Discussion

The infection rate of *T. gondii* still remains greater than 10% in the United States even though there has been a slightly decline in seroprevalence of *T. gondii* from 13.2% in 2009–2010 [18] to 11.14% in 2011–2014 [19]. On the other hand, the prevalence of NAFLD is as high as 30% in the same population [20, 21]. Our study showed a higher incidence of NAFLD in the *T. gondii* seropositive group than in the seronegative group (27.10% vs 23.40%, *p* < 0.001). However, multivariate regression indicated that infection might not be an independent risk for NAFLD.

Evidence from basic and clinical studies indicated the *T. gondii* infection might be responsible for liver steatosis [7, 8]. A significant change in pathology has been found in mice infected with *T. gondii*, including inflammatory cell infiltration, hepatocyte necrosis and hepatosteatosis [10]. Further transcriptomic analysis of *T. gondii* infected mice showed downregulation of peroxisome proliferator-activated receptors signaling pathway in the liver [10], which has long been proven to play key roles in regulating host bile biosynthesis, fatty acid metabolism, lipid metabolism and energy metabolism [22]. Besides the direct influence in the liver, *T. gondii* infection may play a role in diabetes mellitus, which is a well-known risk factor of NAFLD [23–25]. *T. gondii* infected mice had a significant reduction of pancreatic islet cells, as well as an apparent decrease in insulin expression [23]. The results from this study also demonstrated higher BMI, cholesterol, triglyceride, uric acid and FBG levels in *T. gondii* seropositive group, also suggesting an effect of *T. gondii* infection on metabolism. All these mechanisms might contribute to the higher NAFLD levels seen in the *T. gondii* antibody seropositive population.

However, in multivariate analysis, *T. gondii* infection was not an independent factor for NAFLD. Unmeasured confounders may explain the association between NAFLD and *T. gondii*. Although basic and clinical studies had shown an increased risk of NAFLD in *T. gondii* infected patients, the results of this study found that, rather than *T. gondii* infection, only age, gender, BMI, uric acid, fasting blood-glucose and serum lipid levels were independent risk factors for the presence of NAFLD. This might be because *T. gondii* infection alone is a confounder which is associated with other NAFLD-related variables, such as obesity, diabetes or hyperlipidemia [25–27]. The prevalence of *T. gondii* rises with age [18, 19]. As shown in Fig. 1c, the incidence of NAFLD also increases with age, regardless of the presence of *T. gondii* antibody. It is possible that the positive correlation between NAFLD and *T. gondii* is indirect, and that age plays a key role in it. These questions require further exploration through basic research.

However, the direction of the causal relationship between NAFLD and T. gondii infection might be opposite. Based on this cross-sectional study, we could not rule out the possibility that NAFLD might increase the risk of *T. gondii* infection. Patients with chronic liver diseases, including NAFLD, are susceptible to various pathogens infection [8]. Those patients have a depressed immune response in both cell-mediated and humoral immunity [20]. As a result, they might have a remarkably declined ability to protect the hosts against T. gondii infection. Several researches have shown a higher seroprevalence of *T. gondii* in patients with chronic liver diseases than those without in China and Egypt [7, 8]. In this study, the NAFLD patients also have an increasing *T. gondii* seropositive rate(29.6% vs. 25.7%, *p* < 0.001, data not shown). Those studies, including the present one, suggest the possibility that NAFLD is a risk factor for *T. gondii* infection cannot be excluded. However, a small-sized study from Mexico including 75 adults with liver disease (approximately 50% were alcoholic liver diseases) and 150 controls failed to demonstrate a relationship between seroprevalence of anti- *T. gondii* antibodies and liver disease [28]. Therefore, the complex relationship between *T. gondii* and NAFLD requires further research.

The limitation of this study was that data were collected from 1988 to 1994 when the prevalence of *T. gondii* was different from recent years. However, as far as we know, it was the largest population-based investigation containing datasets of both NAFLD and anti- *T. gondii* antibodies. Nevertheless, our results clarify the relationship between *T. gondii* infection and NAFLD to some extent and offer interesting and useful evidence to further the understanding of both diseases.

## Conclusions

In conclusion, patients with *T. gondii* infection have a higher prevalence of NAFLD. Age may have an effect on the increase of NAFLD in the *T. gondii* seropositive population.

## Abbreviations

ALP: Alkaline phosphatase
ALT: Alanine transaminase
AST: Aspartate aminotransferase
FBG: Fasting blood-glucose
GGT: Gamma-glutamyl transpeptidase
HBA1c: Glycated hemoglobin
TBIL: Total bilirubin
